# A national multidisciplinary healthcare network for treatment of hepatitis C in people who inject drugs in Slovenia

**DOI:** 10.1186/1471-2334-14-S6-S6

**Published:** 2014-09-19

**Authors:** Mojca Maticic

**Affiliations:** 1 Clinic for Infectious Diseases and Febrile Illnesses, University Medical Centre, Ljubljana, Slovenia

## 

Slovenia’s estimated 1%–2.5% hepatitis C virus (HCV) seroprevalence rate in the general population is much lower than the 3.5% rate reported by neighbouring Italy [1]. There are an estimated 10,000 people who inject drugs (PWID) in Slovenia, a country of two million. HCV seroprevalence in PWID in Slovenia has been estimated at 23% – the second-lowest rate in the European region – and HIV co-infection is extremely rare [2,3]. Around 4,500 PWID per year receive services at regional Centers for the Prevention and Treatment of Drug Addiction (CPTDA), with around three-quarters receiving opioid substitution treatment [4]. In 2006, HCV RNA prevalence among 1,450 PWID managed at CPTDA was 15.6%; 1% of the infected cohort had completed HCV treatment and 3% were currently receiving it [5].

In Slovenia, the relatively low HCV seroprevalence in PWID most probably reflects the systematic introduction of various well-established harm reduction interventions in the early 1990s [4]. At the beginning of the last decade, however, the HCV treatment rate for PWID was reported to be very low, as was the case in other European countries [6]. To increase the HCV treatment rate, a national model for the management of HCV infection in this vulnerable population was introduced.

HCV is managed by two institutions in Slovenia: the National Institute of Public Health of the Republic of Slovenia and the National Viral Hepatitis Expert Group, the latter a multidisciplinary body of experts from various public medical institutions. The National Institute of Public Health is responsible for national epidemiological surveillance and for HCV prevention strategies such as blood safety, vaccination and contact tracing. The National Viral Hepatitis Expert Group, which was founded by a self-initiative in 1997, guides the overall national strategy for the management of viral hepatitis, drawing on best practices and standards of care as much as possible. Case finding in special groups, anonymous testing, treatment, national consensus guidelines, research, education and mass media campaigns are all issues that fall within the Expert Group’s domain [7].

Slovenia formulated national guidelines for treatment of HCV infection in 1997, basing the guidelines on international recommendations while taking into account national particularities [7]. Treatment in Slovenia is fully publicly funded and is provided with no limitations except for one: it must be prescribed by viral hepatitis specialists at one of five hospital-based clinics in accordance with national guidelines. The 1997 guidelines did not exclude PWID from HCV treatment, stating that treatment decisions should be made on an individual basis. Since 1997, the Expert Group has systematically monitored treatment efficacy and safety in Slovenian patients treated for hepatitis C.

In 1995, 18 CPTDA were established in Slovenia. When a PWID enters a CPTDA programme, the person is offered HCV testing. Individuals who test negative are invited to be tested again every six to 12 months [4]. To increase the proportion of PWID treated for HCV and to achieve optimal treatment adherence, efficacy and safety, a multidisciplinary national healthcare network for the treatment of HCV infection in PWID was established in 2007 [8]. This network linked the 18 CPTDA and the five specialised hosptial-based clinics for treatment of viral hepatitis. The Clinic for Infectious Diseases and Febrile Illnesses at the University Medical Centre in Ljubljana serves as the reference institution.

The network for the treatment of HCV in PWID includes clinical care providers (addiction therapists and viral hepatitis specialists, most of whom are infectologists), counsellors (nurses and social workers), and psychiatrists who have acquired additional medical education and supportive training. It also includes peers and other members of patient support systems such as family members, friends and co-workers. Collaboration among all involved health professionals takes place through participation in national conferences that have been held annually since 2006.

Slovenian national consensus guidelines for the management of HCV infection in drug users were established in 2007 [8]. These guidelines set forth best practices for the comprehensive management of HCV-infected PWID. They provide best practices for screening and identifying treatment-eligible PWID, as well as for providing them with high-quality HCV education, counselling and motivation. Best practices for treatment of HCV in clinical settings for viral hepatitis are provided, with at least monthly interventions performed individually throughout the treatment period in close cooperation between viral hepatitis specialists and addiction therapists.

Before the introduction of the multidisciplinary model, PWID made up the following proportions of all patients treated for HCV in Slovenia: 5% in 1997–1999, 16% in 1999–2001 and 36% in 2002–2004 [9-11]. In 2008–2010, this proportion increased to 78%. It is notable that by the end of 2010 approximately 13% of HCV-infected PWID at CPTDA had already received treatment. The sustained virological response among PWID was 80% in the last monitoring period [11].

In conclusion, the multidisciplinary healthcare network enables individual evaluation and management of HCV-infected PWID by both drug addiction therapists and viral hepatitis specialists in a convenient, experienced and effective manner in the context of Slovenian circumstances. Since healthcare systems and settings vary greatly across Europe, each European country should try to develop its own interdisciplinary model for addressing the treatment of HCV in PWID.

## Competing interests

The authors declare that they have no competing interests.

## Declarations

This article has been published as part of *BMC Infectious Diseases* Volume 14 Supplement 6, 2014: Viral Hepatitis in Europe. The full contents of the supplement are available online at http://www.biomedcentral.com/bmcinfectdis/supplements/14/S6. The publication charges for this supplement were funded by AbbVie as an unrestricted grant to Rigshospitalet, the University of Copenhagen. AbbVie further funded the printing of the supplement with additional financial support from the Drug Prevention and Information Programme (DPIP) of the European Union.
